# The Venom Proteome and Immunorecognition Profile of Clinically Important *Echis carinatus sochureki* from Northwestern India Underscores the Need for Regionally Specific Antivenoms

**DOI:** 10.3390/toxins18010054

**Published:** 2026-01-21

**Authors:** Akhilesh Kumar, Alka Sahu, Maya Gopalakrishnan, Avni Blotra, Vishal Kumar Rout, Sourish Kuttalam, Shibi Muralidar, Anita Malhotra, Karthikeyan Vasudevan

**Affiliations:** 1 All India Institute of Medical Sciences, Jodhpur 342005, India; akhileshph@gmail.com (A.K.); maya.gopalakrishnan@gmail.com (M.G.); 2 CSIR-Centre for Cellular and Molecular Biology, Hyderabad 500007, India; alkasahuak@gmail.com (A.S.); avniblotra76@gmail.com (A.B.); vishalrout3131@gmail.com (V.K.R.); shibi3214@gmail.com (S.M.); 3 Academy of Scientific and Innovative Research (AcSIR), Ghaziabad 201002, India; 4 Molecular Ecology and Evolution at Bangor, School of Environmental and Natural Sciences, Bangor University, Bangor LL57 2UW, UK; bsu8d2@bangor.ac.uk; 5 Captive & Field Herpetology Ltd., 13 Hirfron, Llaingoch, Holyhead LL65 1YU, UK; 6 Society for Nature Conservation, Research and Community Engagement (CONCERN), Nalikul 712407, India

**Keywords:** Viperidae, geographic variation, reverse-phase liquid chromatography, mass spectrometry, intraspecific comparison, coagulopathy, cross-neutralization

## Abstract

The saw-scaled viper *Echis carinatus*, one of the “Big Four” causes of snakebites in India, is found from Sri Lanka to eastern Iraq. To investigate clinical reports regarding the limited efficacy of Indian polyvalent antivenom (IPAV) against envenomation in *Echis carinatus sochureki* (ECS) in northwestern India, we obtained 22 snakes from three locations in Rajasthan and identified 148–174 toxin isoforms belonging to 21–25 toxin families in their venom using a bottom-up proteomics approach. All samples showed a high abundance of snake venom metalloproteinases (SVMPs), particularly SVMP class III. Other major components were phospholipases A_2_, L-amino-acid oxidases, snake venom serine proteases and snaclecs (C-type lectins). Variation in venom composition among locations in Rajasthan, compared to *E. c. carinatus* (ECC) from southern India, was primarily due to differences in the relative abundance of these toxin families. Recognition of all venom components by IPAV was poor at lower antivenom concentrations. Notably, SVMP classes II and III were poorly recognized at all venom-to-antivenom ratios in all ECS venoms, and a plasma clotting assay revealed poor neutralization of procoagulant activity. This collaborative study highlights the need for the development of regional antivenoms to effectively treat snakebites in northwestern India.

## 1. Introduction

While snake venom has not evolved to target humans, accidental envenomation is a manifest and serious public health issue. Snake venoms comprise a diverse mixture of proteins (>95%), carbohydrates, lipids, nucleosides, salts, and amino acids [1]. The protein content in these venoms reportedly varies through the proteoforms, relative abundance, and biological functions across geography [2], ontogeny [3], and species [4]. Snake venom is an adaptation to immobilize prey and also serves to defend snakes against threats [5]. The Indian sub-continent stands as a global hotspot of snakebites, with an estimated more than one million snakebite incidents occurring annually, resulting in over 51,100 deaths (95% UI 29,600–64,100) and also leaving many survivors with severe disabilities and morbidities [6]. The majority of these snakebite-related deaths are attributed to the Indian “Big Four” species: *Daboia russelii*, *Echis carinatus*, *Bungarus caeruleus*, and *Naja naja*. However, with a large diversity of snakes in the region, about 60 species of which are venomous and are known to cause envenomation in humans [7], there is a pressing need to shift the focus beyond the Big Four.

The only available treatment for human envenomation is Indian polyvalent antivenom (IPAV), which contains F(ab)_2_ fragments purified after hyper-immunizing equines against the venom of the Big Four species. However, venoms for immunization are currently sourced exclusively from the Irula Snake Catchers’ Industrial Cooperative Society in Tamil Nadu [8] located on the southeastern coast of India. This centralized collection overlooks the extensive geographical variation in venom composition among medically important snakes across India. Multiple studies have suggested that IPAV may be less effective or even ineffective in non-source populations and often lacks the necessary antibodies to neutralize many crucial toxin components [9,10,11,12].

The saw-scaled viper, one of the Big Four venomous snakes, is represented by two subspecies: *Echis carinatus carinatus* (ECC) and *Echis carinatus sochureki* (ECS), although their exact distribution has not yet been determined. Furthermore, mortality and morbidities caused by the subspecies ECS are a major, yet under-reported, public health issue in northwestern India and southern Pakistan [13,14]. The primary clinical manifestations of ECS envenomation are edema, venom-induced consumption coagulopathy (VICC) resulting in bleeding, acute kidney injury, and local tissue complications such as blistering and hemorrhage [15]. Despite administration of the maximum recommended dose of IPAV, clinical outcomes remain poor [15,16,17]. Clinical reports have documented life-threatening VICC, prolonged hospital stays, intensive care admissions, excessive use of IPAV, and increased requirement of blood products, highlighting the limited clinical effectiveness of IPAV for ECS envenoming. Apart from these clinical reports, the proteomic and transcriptomic studies on ECS and ECC suggest notable differences in the venom composition [18,19]. These factors might be responsible for the poor neutralization by IPAV.

**Figure 1:**
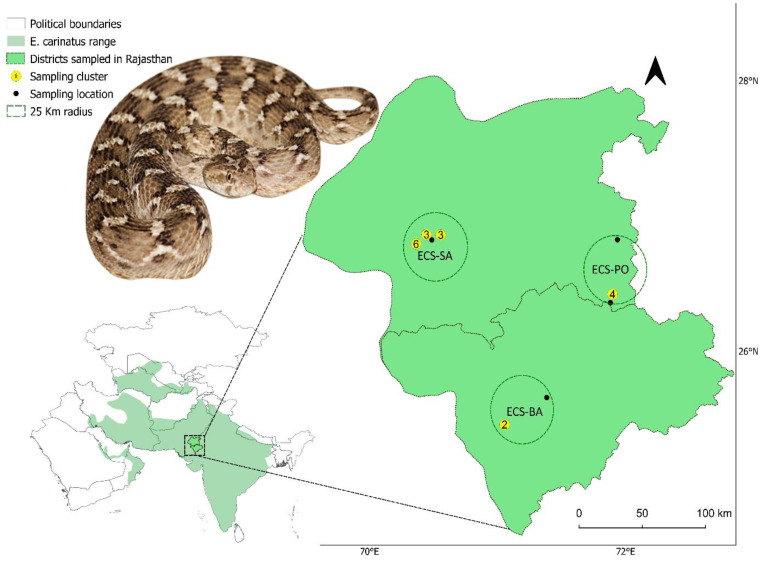
Geographical distribution of *Echis carinatus* as indicated in the spatial distribution database of IUCN (https://www.iucnredlist.org/ accessed on 6 November 2025). The darker shade of green highlights the two districts of Rajasthan from which *Echis carinatus sochureki* venom was collected. Yellow numbered dots indicate the cluster of individuals sampled within a 1 km range. Black dots indicate the sampling location of individuals beyond that proximity. The circles around ‘ECS-SA’, ‘ECS-BA’, and ‘ECS-PO’ indicate a 25 km radius and denote the set of individual samples pooled to generate three region-specific venom pools representing Sam (ECS-SA), Barmer (ECS-BA), and Pokhran (ECS-PO) of Rajasthan, India. Inset photo by A.S.

Based on these prior studies, we hypothesized that ECS venom exhibits significant biochemical and antigenic differences from ECC venom, and that these differences contribute to the reduced recognition of venom toxins by immunoglobulins present in IPAV. To test this, in this study, we aimed to characterize the venom proteome of ECS venoms from the arid regions of Rajasthan and to evaluate the neutralization and immunorecognition efficacy of Indian polyvalent antivenom (IPAV) against these venoms in comparison to ECC venom. By integrating proteomics, second-generation antivenomics, and functional assays, we provide the detailed venom proteomes and compositional differences of ECS from three different locations in the Thar Desert (Figure 1), identify snake venom metalloproteases (SVMP) as key toxins that escape immunorecognition, and quantify the extent of IPAV binding and neutralization. Given the medical significance of envenomation caused by *E. c. sochureki* [15,16,17,20], these findings have the potential to improve the clinical management of ECS snakebites in the region.

## 2. Results

### 2.1. Sodium Dodecyl Sulfate-Polyacrylamide Gel Electrophoretic Analysis of Venoms

The protein bands in both reducing and non-reducing conditions revealed a conserved core set of protein bands represented in all the samples. The molecular weights of the protein bands ranged from ~210 kDa to ~6.5 kDa. Low-molecular-weight (<30 kDa) bands were more distinct in the reducing conditions than in the non-reducing conditions. Consistent prominent bands were observed around ~55 kDa and ~25–30 kDa, and diffuse bands at ~10–18 kDa; they may correspond to SVMP-III and L-amino acid oxidases (LAAO), P-II SVMP, and phospholipase A_2_ (PLA_2_) isoforms, respectively. Some inter-individual variation was observed, particularly in the intensity and presence of low-molecular-weight bands. ECS-SA:23.50 was the only sample that exhibited a distinct elution pattern from the other ECS samples (Figure S1).

### 2.2. Reverse-Phase High-Performance Liquid Chromatography Separation (RP-HPLC)

**Figure 2:**
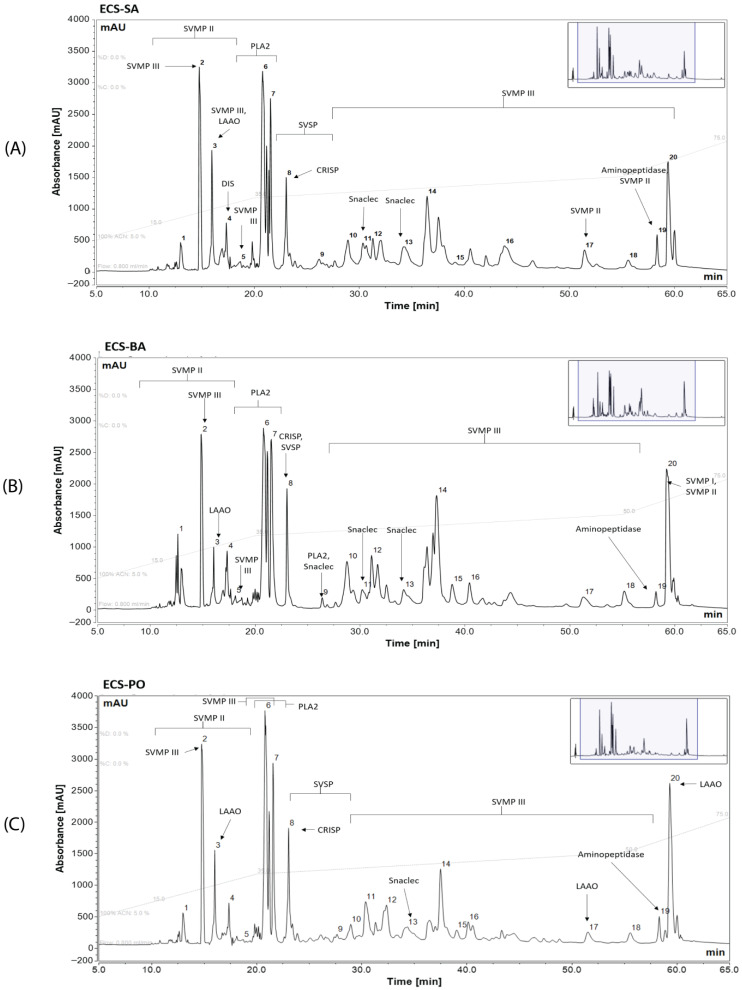
Reverse-phase chromatograms of pooled venoms of *E. c. sochureki* showing the elution profile of venom proteins in (**A**) ECS-SA (*n* = 13), (**B**) ECS-BA (*n* = 3), and (**C**) ECS-PO (*n* = 6), respectively. The grey dotted lines indicate the acetonitrile gradient during the run. Annotations indicate the major components eluting in the peak, and the numbers correspond to the peak fractions collected during the RP-HPLC fractionation. Abbreviations: SVSP—Snake venom serine proteinases, SVMP II, III—Group P-II and P-III Snake venom metalloproteinases, PLA_2_—Phospholipase A_2_, LAAO—L-amino acid oxidases, CRISP—Cysteine-rich secretory proteins.

RP-HPLC fractionation of ECS venom resulted in approximately 20 distinct peaks across a 15–75% acetonitrile (ACN) gradient. The pooled groups from Sam (ECS-SA), Barmer (ECS-BA), and Pokhran (ECS-PO) displayed broadly similar elution profiles, with the most intense peaks eluting in decreasing order of peak fraction numbers 2, 6, and 7 for ECS-SA, and 6, 2, and 7 for ECS-BA and ECS-PO, occurring between 18 and 36% ACN (Figure 2). This suggests a conserved set of highly abundant proteins across regions. Population-specific differences were also observed in the 35–55% ACN range, with peak fractions 1, 14, and 15 being notably elevated in ECS-BA compared to ECS-SA and ECS-PO, indicating an increased representation of moderately hydrophobic proteins. Additional variability was observed in peak fractions 9–13, 16, and 20, involving both the number of discernible peaks and their relative intensities across the three venoms.

Peak annotation further underscored differences among the venom profiles. Peak fractions 1 to 3 in all venoms were dominated by P-II and P-III SVMPs and LAAO. Notably, LAAO was enriched in ECS-SA and ECS-PO more so than in ECS-BA. Fraction 4 contained PII SVMPs, and fractions 4 to 7 were primarily composed of PLA_2_s, with an additional PIII-SVMP contribution in ECS-PO. Cysteine-rich secretory proteins (CRISPs) and snake venom serine proteinases (SVSPs) appeared consistently in fractions 8 to 9 across all samples. Fractions 10–16 were richer in PIII-SVMPs. Snaclecs showed minor variation in their respective peak areas; however, higher signal intensities in fractions 14–15 were observed in ECS-BA. In the later fractions (17–20) of all venoms, PI, PII, PIII-SVMPs, LAAO, and aminopeptidases were detected. Among the three populations, ECS-SA showed a more diverse representation of toxin types in these fractions. Collectively, these results indicate a shared toxin framework with notable differences among populations in the distribution and relative abundance of SVMPs, PLA_2_s, and secondary venom components.

### 2.3. Venom Composition

A database search against the Viperidae identified 210 proteins for ECS-SA, 156 for ECS-BA, and 180 for ECS-PO, which were classified into 11, 9, and 12 toxin families, respectively.

In ECS-SA, the venom was composed predominantly of PIII-SVMPs (60.96%), followed by PLA_2_ (12.35%), PII-SVMPs (5.67%), and LAAO (5.49%). Minor components included SVSPs (3.95%), snaclecs (3.64%), disintegrins (DIS) (2.2%), aminopeptidase (1.86%), and CRISPs (1.43%).

In ECS-BA, PIII-SVMPs dominated 52.67% of the total composition, but notably, PLA_2_ (26.96%) was more than twice as abundant as in ECS-SA and ECS-PO. PII-SVMPs and LAAO each accounted for 5.47% and 5.99%, respectively, and PI-SVMPs for 2.52%, while CRISPs (1.98%) and SVSPs (1.39%) were present at lower levels. Snaclec (0.97%) and aminopeptidase (1.27%) were minor components and DIS (0.04%) was nearly absent.

In ECS-PO, PIII-SVMPs remained the most abundant (41.59%), yet at the lowest proportion among the three populations. LAAO was highest in this group (15.11%), while PLA_2_ abundance was lowest (12.49%) compared to the other two proteomes. Other toxin families included PII-SVMPs (13.02%), SVSPs (7.52%), snaclec (2.64%), CRISPs (2.33%) and DIS (0.63%). Interestingly, SVMP inhibitors (0.86%) were uniquely detected in this sample.

**Figure 3:**
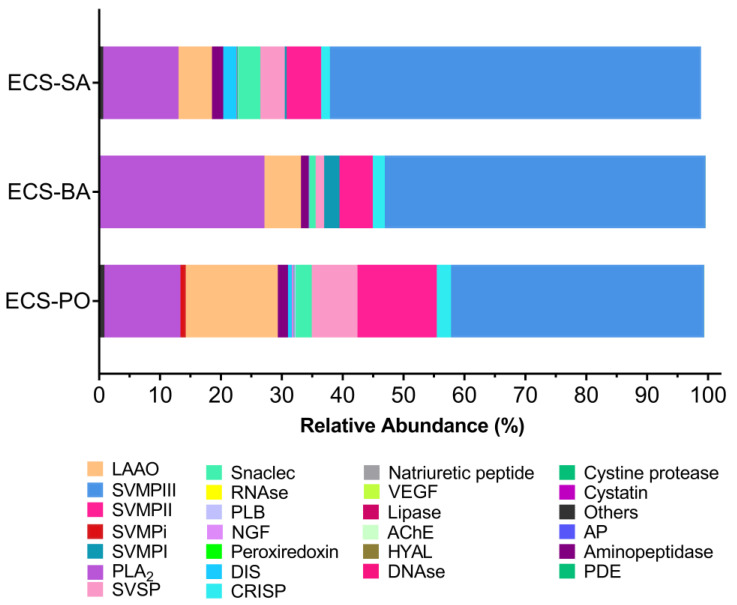
Bar plot showing the relative abundance of toxin families in the pooled venoms ECS-SA, ECS-PO and ECS-BA. Abbreviations: VEGF—Vascular endothelial growth factor, SVSP—Snake venom serine proteinases, SVMPI, II, III—Group PI, PII, and P-III Snake venom metalloproteinases, SVMPi—Snake venom metalloproteinase inhibitor, PLB—Phospholipase B, PLA_2_—Phospholipase A_2_, PDE—Phosphodiesterase, LAAO—L-amino acid oxidases, HYAL— Hyaluronidase NGF—Nerve growth factor, DIS—Disintegrins, AchE—Acetylcholine-esterases, CRISP—Cysteine-rich secretory proteins.

For these three site-specific profiles, SVMPs were the predominant toxin family, with PIII-SVMPs as the major subclass in all. PLA_2_ constituted the second major toxin family, being most abundant in ECS-BA, followed by ECS-PO and ECS-SA. ECS-PO showed comparatively higher levels of LAAO and SVSP than those from ECS-SA and ECS-BA. Minor components such as CRISP, snaclec, and aminopeptidase also varied across sites. Snaclecs were more abundant in ECS-SA and least represented in ECS-BA, whereas CRISP levels were slightly higher in ECS-PO compared to ECS-SA and ECS-BA. Overall, the venom profiles revealed both conserved features and variation in toxin composition, reflected through differences in the relative abundance of major and minor toxin families among the three sampled groups (Figure 3, Table S1).

### 2.4. Affinity Chromatography

#### 2.4.1. ECS-SA

**Figure 4:**
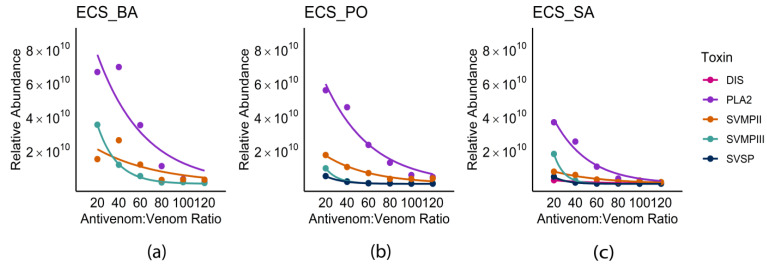
Trend in relative abundance of dominant toxins in non-retained fractions with increasing antivenom–to–venom ratio in (**a**) ECS-BA, (**b**) ECS-PO, and (**c**) ECS-SA.

At an antivenom–to–venom ratio of 20:1, the RP-HPLC analysis of the non-retained fractions (Figure S2) revealed major peaks corresponding to SVMP-II, SVMP-III, LAAO, DIS, and PLA_2_, compared to control profiles. Quantitative MS/MS analysis showed PLA_2_ as the most abundant component (3.65 × 10^10^), followed by SVMP-III (1.77 × 10^10^), SVMP-II (7.21 × 10^9^), SVSP (4.18 × 10^9^), and DIS (2.15 × 10^9^), with additional minor toxin families including LAAO, aminopeptidase, SVMP-I, and snaclec. Increasing the antivenom–to–venom ratios (20:1 to 120:1) resulted in a sequential decrease in the relative abundance of SVMP-III and PLA_2_, followed by SVSP. In contrast, no substantial change in the neutralization of DIS and SVMP-II was detected (Figure 4a).

#### 2.4.2. ECS-BA

At an antivenom–to–venom ratio of 20:1, the RP-HPLC analysis of the non-retained fractions similarly showed peaks corresponding to SVMP-II, SVMP-III, and PLA_2_ (Figure S3). Quantitative MS/MS analysis further identified PLA_2_ (6.62 × 10^10^), SVMP-II (1.47 × 10^10^), and SVMP-III (3.5 × 10^10^) as the dominant unbound components. The abundance of SVMP-III and PLA_2_ exhibited a sharp decline with increasing antivenom–to–venom ratios, showing ~50% reduction at 60:1 and near-complete binding at a 120:1 ratio. SVMP-II showed comparatively less binding with the antivenom, even at higher antivenom–to–venom ratios. Minor toxin families detected at ratios above 20:1 (LAAO, SVMP-I, and DIS) were effectively neutralized at low antivenom–to–venom ratios (Figure 4b).

#### 2.4.3. ECS-PO

Non-retained fractions similarly showed peaks corresponding to SVMP-II, SVMP-III, and PLA_2_ (Figure S4). At the 20:1 antivenom–to–venom ratio, both RP-HPLC and qMS/MS identified PLA_2_ (5.55 × 10^10^), SVMP-II (1.71 × 10^10^), SVMP-III (9.22 × 10^9^), and SVSP (4.61 × 10^9^) as the predominant toxin families. The binding of SVSP and SVMP-II reached saturation at 60:1 and 80:1 antivenom–to–venom ratios, respectively, whereas SVMP-III showed only a modest decline between 60:1 and 120:1. PLA_2_ showed a progressive decrease in relative abundance across all ratios. Minor toxin families, including LAAO, snaclec, and aminopeptidase, were consistently detected at low abundance across all antivenom concentrations (Figure 4c).

### 2.5. Clotting Activity

**Figure 5:**
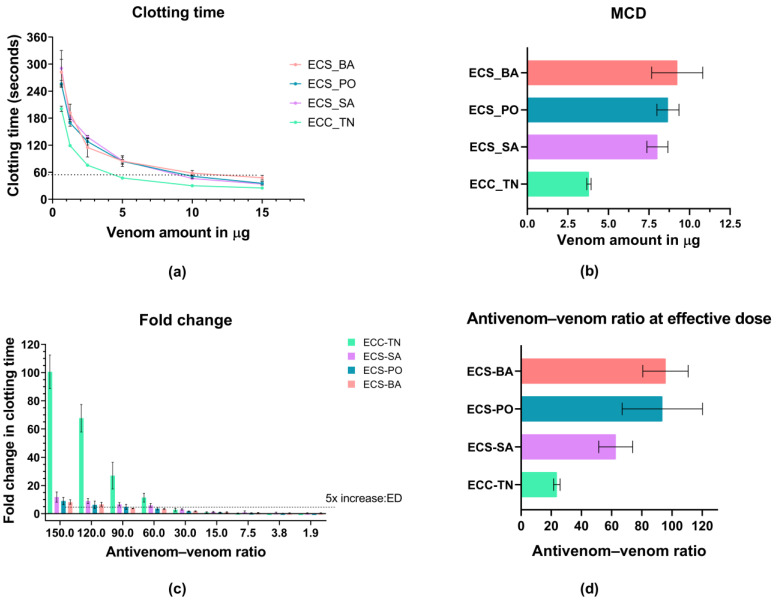
Neutralization of clotting activity of *Echis carinatus* venoms by IPAV. Values represent mean ± SEM from three independent technical replicates per venom sample. ECS venoms were pooled from ECS-SA (*n* = 13), ECS-PO (*n* = 6), and ECS-BA (*n* = 3) individual snakes. ECC-TN is a commercially sourced reference venom for which sample size information is not available. (**a**) Clotting times (CT) for *E. c. carinatus* (ECC-TN) and *E. c. sochureki* (ECS-BA, ECS-PO, ECS-SA) across different venom amounts. (**b**) Bar plot showing minimum clotting dose (MCD) values. MCD for ECC-TN (3.8 ± 0.1 µg) was significantly lower than for ECS venoms (8.0–9.2 µg) (one-way ANOVA, F(3,8) = 21.69, *p* < 0.01). (**c**) Fold changes in CTs at increasing antivenom–venom ratio with a marked rise beyond 25:1. Dotted line marks the 5-fold increase in CT. (**d**) Bar plot representing effective dose (ED) ratios (antivenom–to–venom ratios) required for five-fold CT prolongation were significantly lower for ECC-TN (23.7 ± 1.2) than for ECS-BA (95.7 ± 8.7), ECS-PO (93.6 ± 15.4), and ECS-SA (62.7 ± 6.5) (ANOVA, F(3,8) = 12.80, *p* < 0.01).

The clotting times (CT) for venoms are shown in Figure 5a (Table S2). ECC-TN showed the lowest minimum clotting dose (MCD) (3.80 ± 0.08 µg), while higher values were observed for ECS-BA (9.25 ± 0.91 µg), ECS-PO (8.67 ± 0.40 µg), and ECS-SA (8.02 ± 0.37 µg) (Figure 5b). One-way ANOVA revealed significant differences in clotting activity and MCD among the venom groups (F(3,8) = 21.69, *p* = 0.0003). Post hoc Dunnett’s comparisons confirmed that all three venoms had significantly higher MCDs than ECC-TN (adjusted *p* < 0.01) (Table S3). The clotting observed was probably induced by heparin-insensitive prothrombin activators and thrombin-like enzymes in these venoms. As the MCD values were different, the amount of antivenom required varied across groups to maintain consistent antivenom–to–venom ratios.

Across all groups, a marked increase in clotting time and fold change was observed at antivenom–to–venom ratios greater than 25:1 (Figure 5c, Table S4). The effective dose (ED), taken as the ratio of antivenom that prolonged CT by five-fold, also differed significantly among groups (One-way ANOVA; F(3,8) = 12.80, *p* = 0.002) (Table S5). The lowest ED ratio was observed for ECC-TN (23.7 ± 1.2), while significantly higher ratios were observed for ECS-BA (95.7 ± 8.7), ECS-PO (93.6 ± 15.4), and ECS-SA (62.7 ± 6.5). Dunnett’s post hoc analysis confirmed that ECC-TN differed significantly from all other groups (adjusted *p* < 0.05), with the largest differences observed against ECS-BA and ECS-PO (Figure 5d, Table S6).

## 3. Discussion

Envenomation caused by *E. c. sochureki* has long been recognized as extremely challenging by the clinicians in northwestern India [15,16]. However, the reasons underlying the observed clinical complications and low efficacy of IPAV have not been previously identified. Through bio-molecular assays, we provide evidence and attempt to fill key knowledge gaps on variations in the venom proteomes within the subspecies. The venom is vasculotoxic and causes VICC [20], acute kidney injury [21], and local complications as the predominant clinical manifestations, suggesting the action of SVMPs, PLA_2_s, SVSPs, and LAAOs. Venoms from all three locations (ECS-SA, ECS-BA, and ECS-PO) had similar RP-HPLC elution profiles, but the relative protein composition and abundance varied. However, all venoms were dominated by SVMPs, followed by PLA_2_, SVSPs, and LAAOs. SVMPs were present in multiple RP-HPLC fractions, suggesting the presence of multiple isoforms of the toxin in the venoms. SVMP is also a toxin family that is poorly neutralized by IPAV. SVMPs occur as structural variants (PI, PII, and PIII), each exhibiting diverse functions, including haemorrhagic effects, fibrinogenolytic effects, factor X, prothrombin activation, and inhibition of platelet aggregation. Similarly, SVSPs are thrombin-like enzymes and show fibrinogenolytic activities analogous to thrombin and are typically found in viperid venoms [22]. Clinical observations made on envenomed patients from northwestern India underscore the role of SVMP-dominated venom pathophysiology [23]. Specifically, the observed prolonged hypofibrinogenemia persisting >7 days in ECS envenoming resulting in delayed bleeding and hematomas [15] might be explained by possible fibrinogenolytic activity of SVMPs [23] and SVSPs [24].

The previously reported ECC venom proteome from Rajasthan [9] was based on a pooled sample of ten individual venoms, but specific collection sites were not mentioned. Their analysis showed a predominance of PLA_2_ (61.9%), followed by LAAO (12.6%), SVSPs (7%), and SVMPs (3%). In contrast, our proteomes showed marked compositional differences. The venom proteomes from this study aligned with proteomes of the species that were reported from Iran [25] and with the transcriptomics evidence reported from the UAE [26]. These differences could possibly be attributed to differences in the sample locations, workflow used for data acquisition, the databases used for proteome annotation, and regional or temporal variation in the venom samples. In addition, because individuals from the ECS-BA population were consistently smaller than those from the other regions in our study, ontogenetic influences on venom composition cannot be excluded as a potential contributor to the observed site-specific differences and may constitute a confounding factor. Addressing these will require future studies based on size-matched sampling and individual-level venom analyses prior to population-level comparisons.

A comparison of ECC venoms from Goa, Rajasthan, and Tamil Nadu [2] and ECS venoms analyzed in this study showed SVMPs and PLA_2_ as the dominant toxin families. However, ECC venom from Tamil Nadu had lower SVMP abundance (23.06%) and higher snaclec levels (32.98%) than those in the ECS venoms. Since IPAV is produced using ECC venom from Tamil Nadu, these compositional differences might impose limitations on the efficacy in neutralizing the venom toxins. The neutralization profiles of ECS-SA, ECS-BA, and ECS-PO venoms had similar binding profiles with IPAV. All three venom groups showed pronounced dose-dependent neutralization patterns for PLA_2_ and SVMP-III. This could have resulted from the strong immunogenicity of these toxin families, which results in their antibodies being represented in large amounts in IPAV. SVMP-II and SVSP showed weak interaction with the antivenom, even at the highest antivenom ratios. This could either mean suboptimal antibody representation in IPAV or species-level differences in the toxin epitopes that may have impacted the antigen–antibody interactions. Among the three samples, ECS-SA showed the most rapid and extensive neutralization response, suggesting higher antigen–antibody compatibility, whereas ECS-PO retained detectable levels of metalloproteases and serine proteases even at elevated antivenom concentrations. ECS-BA had partial persistence of SVMP-II and SVMP-III, with the highest antivenom levels. In contrast, LAAO, snaclec, and aminopeptidases remained consistently low in all the ratios and populations, indicating efficient neutralization even at lower antivenom levels.

The requirement of high titres of antivenom for the neutralization of ECS PLA_2_ and SVMP-III suggests that there could be limited antibody affinity and inadequate representation of these toxin isoforms in the immunogen mixture used in IPAV production. Both PLA_2_ [27] and SVMPs [28] are multi-locus gene families that show sequence and functional diversification, leading to substantial structural and antigenic variation across geographically distinct populations. Hence, the antibodies generated from venom from a single location (the vicinity of the Irula Co-operative Society in Tamil Nadu) might result in poor cross-reactivity with geographically distinct venoms, resulting in incomplete or delayed neutralization. While IPAV can partially neutralize the major toxin families, its efficiency is not consistent in different samples. This is also consistent with the clinical observation that the degree of antivenom efficacy varies in western Rajasthan [15]. Representation of a spectrum of toxin variants in the immunogen mixture might address this problem.

It is important to consider our results in the light of the potency declared on the specifications of commercially available IPAV vials, i.e., that 1 mL of commercially available IPAV is expected to neutralize 0.45 mg of ECC venom. A vial of IPAV manufactured by Bharat Serums and Vaccines Ltd. contains 390 mg (95% CI, 155–510 mg) of lyophilized powder, constituting 25.2% or 98 mg (95% CI, 39–125 mg) of protein [29]. At this protein concentration and stated binding efficacy, the antivenom-to-venom ratio approximates to 27:1. However, we found that toxins remained unbound at ratios five times higher than this.

The World Health Organization recommends an initial use of five vials of IPAV for ECC envenomation and ten vials for ECS envenomation treatment [30,31]. Our estimates from second-generation antivenomics suggest that for complete neutralization (at 100:1 or 120:1 ratio) of venom components, five to six times the recommended doses of IPAV may be required, which amounts to ~120 vials. As these observations are solely based on in vitro toxin–antibody interaction data, extrapolation of this dose to clinical dosing regimens is fraught with problems, as the assay does not mimic the pharmacokinetic or pharmacodynamic factors that influence in vivo neutralization [32]. However, clinical reports on ECS envenomation treatment are in accord with our prediction, as the use of an average of 22 vials, and in some cases up to 30 vials, often failed to facilitate patient recovery [20]. Given this scenario, some hospitals in the region sometimes use up to 200 IPAV vials without much clinical benefit [15]. Administration of such high IgG doses poses substantial risks, including anaphylaxis and serum sickness [33].

As antivenomics only reflects the toxin-binding capacity of antivenoms, it is crucial to also assess functional neutralization outcomes. Since VICC represents the predominant clinical manifestation caused by ECS envenomation, neutralization of procoagulant activity provided an essential functional perspective on IPAV efficacy. As ECC venom from Tamil Nadu serves as the immunogen source for the production of IPAV [8], the commercial ECC-TN venom was used as a reference to evaluate neutralization efficacy and regional variation in IPAV potency. The clotting activity and neutralization efficacy of antivenom was significantly different for ECC-TN than for ECS-BA or ECS -SA or ECS-PO. ECC-TN venom also had the lowest MCD, while ECS venoms had high MCD values. This meant that a smaller amount of ECC-TN venom was sufficient to induce clot formation in human blood than was the case for any of the ECS venoms. ECC-TN venom was also effectively neutralized even at a low antivenom-to-venom ratio (23.68:1). The ED ratios were approximately 4.04, 2.65, and 3.95-fold higher in ECS-BA, ECS-SA and ECS-PO, respectively, indicating that the neutralization of procoagulant toxins in ECS venoms required at least three-fold more IPAV than the dose that was required for ECC-TN venom. Given the assay conditions, the relative antivenom efficacy reported here reflects the neutralization of a defined subset of *Echis* venom activity, specifically coagulation mediated by direct prothrombin activators and thrombin-like enzymes. These clotting pathways remain functionally active in anticoagulated or heparinized conditions, unlike the pathways mediated by factors VII, X, or XII. Therefore, antivenom efficacy and relative potency were interpreted within the scope of the defined procoagulant activities. As venom composition can vary among populations, and toxins acting through alternative pathways may be represented at higher abundance, future studies should employ other experimental approaches to capture the full spectrum of the venom bioactivity. Notably, ECS from northwestern India, reaching larger sizes, yielded up to 56 mg of crude venom compared to an average yield of 18 mg in ECC from southern India [11] (Table S7, [34]). This observation is consistent with reports that southern Indian populations of *Echis carinatus* generally attain smaller body sizes [35], and it may help explain the observed differences in antivenom requirements between these populations. The large amounts of venom injected into victims during envenomation, coupled with suboptimal neutralization by IPAV, illuminate the serious challenges faced during clinical management of ECS bites.

## 4. Conclusions

This study has involved experts (field herpetologists, clinicians, molecular biologists, and wildlife protection staff) from multiple disciplines to generate data on an important, yet neglected, public health problem of the region. Such collaborations are needed to address multifaceted challenges like the snakebite burden. The proteome of ECS venoms from northwestern India revealed toxin compositions that were different from that of ECC venom obtained from other regions. SVMP was found to be the dominant toxin in ECS venoms, and second-generation antivenomics showed it was poorly immunorecognized by IPAV. Coagulation assays further corroborated this, with ED values 3.5-fold higher than for ECC. These findings align with clinical reports of poor recovery following ECS envenomation and underscore the need for antivenom strategies that effectively target SVMPs. Using antivenomics, we have been able to identify toxin-specific deficiencies in IPAV binding that provide insights for improvements in antivenoms. Collectively, the findings highlight the need for further investigations to enhance IPAV efficacy in a region-specific manner, with particular emphasis on northwestern India and southern Pakistan. Future efforts should focus on enhancing the potency and specificity of antivenoms by improving immunogen design and the inclusion of regionally relevant venoms in IPAV production systems.

## 5. Materials and Methods

### 5.1. Venom Collection

A total of 22 ECS snakes were captured from the deserts of the Barmer and Jaisalmer districts of Rajasthan (Figure 1) and subsequently released after venom extraction. Extraction of venom was performed by gently massaging the glands, prompting the snakes to bite on the Parafilm membrane fastened onto a Nalgene beaker [36]. The venoms were collected from the beaker, placed into 1.5 mL microcentrifuge tubes, and desiccated using silica beads in vacuum chambers. The dried samples were stored at −80 °C as soon as practicable. ECC-TN venom, obtained from the Irula Snake-catchers Industrial Cooperative Society, Tamil Nadu.

### 5.2. SDS-PAGE

Dried crude venom from 22 specimens of ECS (Table S7) was reconstituted in Milli-Q water (0.5 mg in 50 µL). Protein concentration in the samples was estimated using a Thermo Nanodrop Lite spectrophotometer (ThermoFisher Scientific, Waltham, MA, USA), and 20 µg of each sample was subjected to electrophoretic separation under reducing and non-reducing conditions using 12% SDS-PAGE. A broad range marker (Takara catalog #3597A) was used as a reference. Bands were visualized by Coomassie brilliant blue R-250 staining, followed by destaining in methanol–acetic acid solution. To generate population-level venom profiles, based on the SDS results, samples from each location were pooled in equimolar amounts to form three regional groups (each within a ~25 km radius, see Figure 1): ECS-BA (Barmer, *n* = 3; 0 M, 3 F), ECS-SA (Sam, *n* = 13; 1 M, 12 F), and ECS-PO (Pokhran, *n* = 6; 1 M, 5 F). Snout–vent lengths for each of the three populations ranged from 22.5 to 32.5 cm for ECS-BA, 22 to 62 cm for ECS-SA, and 23.5 to 64.2 cm for ECS-PO. Equal quantities of venom from each sample were used to ensure equimolar representation of toxins in each pooled sample; this also ensured sufficient quantity of venom for further analyses. The pooled samples were lyophilized and stored at −20 °C until further use.

### 5.3. Reverse-Phase High-Performance Liquid Chromatography

For protein fractionation, 800 µg of venom from each of the three regional pools was subjected to RP-HPLC using a Thermo Ultimate 3000 UHPLC system (ThermoFisher Scientific, Waltham, MA, USA) equipped with a Zorbax 300SB-C18 column (4.6 × 150 mm, 3.5 µm; Agilent Technologies, Santa Clara, CA, USA). Prior to injection, samples were centrifuged at ~9400× *g* for 10 min to remove particulates. The separation was carried out using a linear gradient of solvent A (0.1% TFA in water) and solvent B (100% ACN), with some modifications, under the following elution conditions: 5% B for 5 min followed by 5–15% B for 5 min, 15–35% B for 10 min, 35–50% B for 35 min and 50–75% B for 10 min. The flow rate was maintained at 0.8 mL/min, and protein elution was monitored at 215 nm [11,37]. Distinct chromatographic fractions were collected manually and vacuum-dried using Savant™ SpeedVac (ThermoFisher Scientific, Waltham, MA, USA).

### 5.4. In Solution Digestion

For proteomic analysis, a total of 10 µg of venom from each peak fraction was subjected to in-solution trypsin digestion. The proteins were denatured using 50 μL of 6 M Urea in 50 mM Tris-HCl, pH 8.5. To reduce the disulphide bonds in the proteins, 2 μL of 200 mM dithiothreitol (DTT) in 50 mM Tris-HCl was added to the peak fractions, followed by 1 h incubation at room temperature. After incubation, 10 μL of 200 mM iodoacetamide (IAA) in 50 mM Tris-HCl was added to acetylate the reduced cysteine residues, and the sample was then incubated in the dark for 1 h. Any unreacted IAA was then quenched by adding 10 µL of 200 mM DTT, followed by another 1 h incubation in the dark. 350 μL of 1 mM CaCl2 in 50 mM Tris-Cl was then added to reduce the urea concentration. Following the addition of trypsin solution at a ratio of 1:30 (*w*/*w* trypsin–protein), they were incubated at 37 °C for 16–20 h. Formic acid was added to lower the pH to 2–4 to stop trypsin action before desalting [11]. Samples were desalted using C-18 Ziptips (Thermo Fisher Scientific, Waltham, MA, USA), and digested peptides were eluted in 70% ACN, 0.1% TFA. Eluted peptides were vacuum-dried and reconstituted in 5% ACN for nano-LC-MS analysis.

### 5.5. MS/MS Data Analysis

Samples were analysed on a Q-Exactive mass spectrometer (Thermo Fisher Scientific, Waltham, MA, USA; hardware ID- 001999D89836) in conjunction with a nanoflow HPLC system (Easy-nLC 1200, Thermo Fisher Scientific, Waltham, MA, USA). The samples were loaded onto Easy Spray Column PepMap ™ RSLC C18 (3 µm,100 Å, 75 µm × 15 cm) (Thermo Fisher Scientific, Waltham, MA, USA) and spectra were generated in positive ion mode. A 45 min linear gradient of solution A (5% ACN in water containing 0.1% formic acid) and solution B (90% ACN in 0.1% formic acid) at the flow rate of 300 nL/min was applied for eluting peptides as follows: 5–25% B for 25 min followed by 25–60% for 12 min, 60–90% for 5 min, and a final 3 min wash at 90% B. Eluted peptides were analyzed in data-dependent acquisition (DDA) mode with MS scans (range: *m*/*z* 400–1750) at a resolution of 70,000. Charge exclusion criteria were applied to ions with +1, +6 to +8, >+8. MS/MS of the top 10 precursor ions with an isolation time of 15 milliseconds was achieved through higher-energy collisional dissociation (HCD) at 30% normalized collision energy. MS/MS spectra were acquired at a resolution of 17,500 at *m*/*z* 200, within a scan range extending from 200 to 2000 *m*/*z*.

Spectra were analyzed using Proteome Discoverer (PD) v2.2 with the SEQUEST-HT algorithm against the Viperidae database (NCBI TaxID: 8689; 105,645 nr sequences) and a keratin contaminant database (15,154 nr sequences). For enhanced accuracy in protein identification, search parameters included a precursor mass tolerance of 10 ppm and fragment tolerance of 0.02 Da. Carbamidomethylation of cysteine was set as a fixed modification, while methionine oxidation and N-terminal acetylation were considered variables. *Percolator* (PD) was used to regulate the false discovery rates (FDR) of peptides and proteins at thresholds of 1% (strict) and 5% (relaxed). Entries corresponding to contaminant or low/moderate FDR confidence were eliminated, and high confidence proteins supported by a minimum of two unique peptides were taken as valid hits. For each protein, the abundances of associated PSMs were summed to estimate the toxin abundance. Toxin families were assigned to the identified entries according to NCBI annotations, which are curated based on the sequence and protein superfamily characteristics. The relative abundances of toxin families were calculated using Equations 1 and 2, essentially integrating precursor ion intensities with corresponding RP-HPLC peak areas at 215 nm (calculated using Chromeleon 7 software). The HPLC fractions were annotated by identifying toxin families contributing >20% of the total abundance within a peak, thereby reflecting the major components.

(1) $$Precursor\;abundance=\sum_{i=1}^{n}{Relative\;peak\;area}_{i}{\;*\;Precursor\;abundance}_{i\;}\;\;\left(n=number\;of\;RP\;peaks\right)$$

(2) $${Relative\;abundance}_{Toxin\;family}=\frac{\sum {Precursor\;abundance}_{Toxin\;family}\;}{Total\;precursor\;abundance}*100$$

### 5.6. Affinity Chromatography for Measuring the Immunorecognition of IPAV

All 22 ECS venom samples from three locations, Sam, Barmer and Pokhran, were grouped as ECS-SA, ECS-BA and ECS-PO, respectively. Third-generation antivenomics was performed using IPAV from Bharat Serums and Vaccines Limited, as an affinity ligand (Batch No. A18224036). This particular antivenom was chosen as it is routinely supplied in Rajasthan for snakebite treatment. The antivenom was dialyzed overnight at 4 °C, lyophilized, and reconstituted in coupling buffer (0.2 M NaHCO_3_, 0.5 M NaCl, pH 8.3). We assessed the immunorecognition at six antivenom-to-venom ratios (20:1, 40:1, 60:1, 80:1, 100:1, and 120:1). For each ratio, a single Micro Bio-Spin column (Bio-Rad, Hercules, CA, USA) with 35.4 ± 0.1 mg of CNBr-activated Sepharose™ 4B beads (Cytiva, Global Life Sciences Solutions Operations UK Ltd., Little Chalfont, UK) was used. The beads were activated with 10 Matrix Bed Volume (MBV) of 1 mM cold HCl. Columns were then equilibrated with 10 MBV of coupling buffer (0.2 M NaHCO_3_, 0.5 M NaCl, pH 8.3). Eight mg of IPAV dissolved in coupling buffer was added to the equilibrated column and incubated at room temperature for 4 h. Post IPAV incubation, the flow-through was collected to estimate the unbound antivenom and to calculate antivenom coupling yield (ACY = Initial IPAV amount loaded minus Amount of IPAV in flow-through). The remaining active groups were blocked using 2 MBV of blocking buffer (0.1 M Tris-Cl, pH 8.5), incubated for 4 h at room temperature, followed by six rounds of alternative washes with 7 MBV of low pH (0.1 M CH_3_COONa, 0.5 M NaCl, pH 4) and high pH (0.1 M Tris-Cl, pH 8.5) buffer. The columns were then equilibrated with 5 MBV of binding buffer (1× PBS, pH 7.4) and incubated with six different venom amounts. The toxin retention by antivenom was measured by comparing NR and R fractions for different amounts of incubated venom proteins (ECS-BA 53.61–307.6 μg, ECS-PO 55–328 μg, and ECS-SA 50.8–331.65 μg) with the control venom profile. For control, beads were incubated without antivenom in coupling buffer, and the highest venom amounts for all three locations were passed through it. The venom toxins that are not bound to the antivenom were collected as flow-through post-incubation and referred to as non-retained (NR) fractions. The bound toxins were further eluted using 10 MBV of the elution buffer (0.1 M Glycine-Cl, pH 2.7) and brought to neutral pH immediately using 1 M Tris-Cl, pH 9, to yield retained (R) fractions. All fractions were dried using Savant SpeedVac™ Vacuum Concentrators and reconstituted in 120 μL of Milli-Q water. Further, 100 μL of these fractions were loaded for reverse-phase HPLC as described in Section 2.3. The remaining aliquots of NR and R fractions were subjected to in-solution trypsin digestion and MS/MS as described previously. Mass spectrometry data were used to identify the most abundant toxins at the lowest antivenom–to–venom ratio (20:1), and their relative abundances were compared across increasing ratios to track sequential neutralization.

### 5.7. Neutralization of Procoagulant Activity of Venoms

A preliminary coagulation assay was performed following Bhatia et al. [12]. Blood was collected in heparinized tubes from two healthy donors. This assay quantifies heparin-insensitive procoagulant activity of *Echis* venoms, and more specifically, the effects mediated by prothrombin-activating toxins and thrombin-like enzymes. Therefore, this approach may be unsuitable for evaluating hemotoxic venoms with heparin-sensitive procoagulant toxins, and clotting mechanisms sensitive to heparin- antithrombin inhibition. An alternative assay would be required to capture these effects. In the current design, platelet-poor plasma (PPP) was extracted through a standard procedure [38,39]. The plasma clotting of venoms was tested for five dilutions, 15, 10, 5, 2.5, 1.25, and 0.6 μg against 200 μL of heparinized PPP from two donors separately. The clotting time (CT) was measured in triplicate. MCD of venoms was estimated at 60 s. All analyses were performed using GraphPad Prism (version 8.0.2). Different dilutions of antivenoms(150:1, 120:1, 90:1, 60:1, 30:1, 15:1, 7.5:1, 3.25:1, and 1.6:1) were added to a challenge dose of venom equivalent to 2MCD and incubated at 37 °C for 30 min. The fold change in the CT was estimated using the clotting times of venom–antivenom complex (*CTvav*) and the venom control (*CTvenom*) according to the following formula:

(3) $$Fold\;change=\frac{CTvav-CT\;venom}{CT\;venom}$$

ED was estimated as the AV ratio at which clotting time increased by five-fold.

### 5.8. Statistical Analysis

ECC-TN venom was designated as the reference control group, since the IPAV is made using venoms from this source. MCD and ED values of venoms from ECS-PO, ECS-BA, and ECS-SA were expressed as mean ± SEM and compared against ECC-TN venom. For each parameter, a one-way ANOVA was used to test for overall differences among groups. When significant, Dunnett’s post hoc test was applied to determine which venoms differed significantly from the ECC-TN (*p* < 0.05).

## Data Availability

The proteomics data of venom composition and second-generation antivenomics from this study are available in the Dryad repository (DOI: 10.5061/dryad.djh9w0wf0).

## Supplementary Materials

The following supporting information can be downloaded at: https://www.mdpi.com/article/10.3390/toxins18010054/s1, Table S1: Relative abundances of toxin families in the venom proteomes of *ECS.*; Table S2: Minimum clotting dose and clotting times recorded for all venoms with increasing venom amounts; Table S3: Post hoc Dunnett’s multiple comparisons of minimum clotting dose (MCD) values using ECC-TN as the reference venom; Table S4: Prolongation in the clotting time of challenge dose of venom at different antivenom–to–venom ratios; Table S5: Comparative coagulation potency and antivenom neutralization efficiency across *Echis* venoms; Table S6: Post hoc Dunnett’s multiple comparisons of effective dose (ED) ratios required for five-fold prolongation of clotting time using ECC-TN as the reference venom; Table S7: Details of the *Echis c. sochureki* venom samples collected from Rajasthan; Figure S1: SDS-PAGE separation of *E. c. sochureki* venoms under reducing and non-reducing conditions; Figure S2: Reverse-phase HPLC profile of ECS-SA toxins after affinity chromatography; Figure S3: Reverse-phase HPLC profile of ECS-BA toxins after affinity chromatography; Figure S4: Reverse-phase HPLC profile of ECS-PO toxins after affinity chromatography.

## Abbreviations

IPAV—Indian polyvalent antivenom; ECS-SA—*Echis carinatus sochureki*-from Sam; ECS-BA—*Echis carinatus sochureki* from Barmer; ECS-PO—*Echis carinatus sochureki* from Pokhran; ECC-TN—*Echis carinatus carinatus* from Tamil Nadu; VICC—Venom induced consumption coagulopathy; SVMP—Snake venom metalloproteinase; LAAO—L-amino acid oxidase; PLA_2_—Phospholipase A2; SVSP—Snake venom serine proteinase; DIS—Disintegrins; CRISP—Cysteine-rich secretory proteins; Snaclec—Snake C-Type lectin; VEGF—Venom endothelial growth factors; PLB—Phospholipase B; PDE—Phosphodiesterase; NGF—Nerve growth factor; Hyal—Hyaluronidase; AP—Aspartic protease; AChE—Acetyl Cholinesterase; CT—Clotting time; MCD—Minimum clotting dose; ED—effective dose; R—Retained toxins; NR—Non-retained toxins; DTT—Dithiothreitol; ACN—Acetonitrile; RP-HPLC—Reverse-phase high performance liquid chromatography; SDS-PAGE—Sodium dodecyl-polyacrylamide gel electrophoresis; IAA—Iodoacetamide.
