# Supplementary material for: The Venom Proteome and Immunorecognition Profile of Clinically Important Echis carinatus sochureki from Northwestern India Underscores the Need for Regionally Specific Antivenoms
Source: Toxins (Basel). 2026 Jan 21;18(1):54. doi: 10.3390/toxins18010054 (PMC12845857; doi:10.3390/toxins18010054)
Supplement: Supplementary file 1 [file toxins-18-00054-s001.zip › toxins-4044053-supplementary.pdf]

Supplementary figures and tables

The venom proteome and immunorecognition profile of clinically important *Echis carinatus sochureki* from northwestern India underscores the need for regionally specific antivenoms

Akhilesh Kumar [1,†,‡], Alka Sahu [2,3,†], Maya Gopalakrishnan [1], Avni Blotra [2,3], Vishal Kumar Rout [2], Sourish Kuttalam [4,5,6], Shibi Muralidar [2], Anita Malhotra [4,*] and Karthikeyan Vasudevan [2,3*]

Table S1. Relative abundances of toxin families in the venom proteomes of *ECS*

Toxin family	*Relative abundance (%)*		
	**ECS-PO**	**ECS-BA**	**ECS-SA**
**SVMPIII**	41.59	52.67	60.96
**PLA2**	12.49	26.96	12.35
**SVMPII**	13.02	5.47	5.67
**LAAO**	15.12	5.99	5.49
**SVSP**	7.52	1.39	4.01
**Snaclec**	2.64	0.97	3.64
**DIS**	0.63	0.04	2.20
**Aminopeptidase**	1.67	1.28	1.86
**CRISP**	2.33	1.98	1.43
**Others**	0.84	0.15	0.64
**SVMPI**	0.00	2.53	0.31
**PDE**	0.10	0.04	0.10
**PLB**	0.21	0.05	0.08
**NGF**	0.27	0.03	0.03
**AP**	0.02	0.04	0.03
**DNAse**	0.02	0.00	0.02
**HYAL**	0.01	0.00	0.02
**VEGF**	0.03	0.02	0.00
**Cystatin**	0.01	0.00	0.00
**Peroxiredoxin**	0.00	0.00	0.00
**Lipase**	0.00	0.00	0.00
**RNAse**	0.00	0.00	0.00
**Cystine protease**	0.00	0.00	0.00
**AChE**	0.00	0.00	0.00
**Natriuretic peptide**	0.01	0.00	0.00
**SVMPi**	0.86	0.00	0.00

Table S2. Minimum clotting dose and clotting times recorded for all venoms with increasing venom amounts.

Venom amount in µg	Clotting time in seconds											
	ECC-TN			ECS-SA			ECS-PO			ECS-BA		
**15**	24.88	24.48	25.71	31.55	36.8	33.17	31.11	38.72	36.22	42.22	48.36	52.7
**10**	29.92	31.14	29.24	47.25	43.02	48	47.11	56.04	49.52	52.16	58.0	63.99
**5**	48.22	47.39	46.11	89.38	76.71	91.18	79.52	87.71	85.31	72.12	96.0	86.22
**2.5**	77.17	74.82	74.72	134.82	142.38	137.36	122.24	125.44	136.61	96.33	138.42	110.26
**1.25**	122.97	116.9	116.95	178.35	193.7	170.36	171.25	175.91	161.38	171.9	211.0	192.38
**0.625**	207.08	196.16	199.12	272.12	263.53	336.2	247.39	259.47	261.41	254.46	312.0	279.84
**MCD**	**3.8**	**3.9**	**3.7**	**7.3**	**8.4**	**8.45**	**8.0**	**8.6**	**9.4**	**7.6**	**10.7**	**9.5**

Table S3. Post hoc Dunnett's multiple comparisons of minimum clotting dose (MCD) values using ECC-TN as the reference venom

Number of families=1; Number of comparisons per family=3; Alpha=0.05

Dunnett's multiple comparisons test	Mean Diff.	95% CI of diff.	Significant?	Summary	Adjusted P Value			
ECC_TN vs. ECS_PO	-4.8	-7.0 to -2.7	Yes	***	0.0005			
ECC_TN vs. ECS_BA	-5.4	-7.6 to -3.3	Yes	***	0.0003			
ECC_TN vs. ECS_SA	-4.2	-6.4 to -2.0	Yes	**	0.0013			
Test details	Mean 1	Mean 2	Mean Diff.	SE of diff.	n1	n2	q	DF
ECC_TN vs. ECS_PO	3.8	8.6	-4.8	0.7	3	3	6.4	8
ECC_TN vs. ECS_BA	3.8	9.2	-5.4	0.7	3	3	7.2	8
ECC_TN vs. ECS_SA	3.8	8.0	-4.2	0.7	3	3	5.6	8

Table S4. Prolongation in the clotting time of challenge dose of venom at different antivenom to venom ratios.

Clotting time in seconds

**Antivenom to venom ratio**	ECC-TN			ECS-SA			ECS-PO			ECS-BA		
**150:1**	4590.47	2838.2	3421.75	351.35	725.41	544.32	396.88	557.73	538.85	281.94	412.66	326.52
**120:1**	2376.79	2186.28	2635.31	313.55	509.11	429.4	297.73	450.23	331.55	212.47	356.51	287.26
**90:1**	990.94	659.05	1304.21	301.11	286.24	367.97	246.43	343.13	273.2	145.06	189.34	194.07
**60:1**	536.19	283.05	506.33	278.45	241.12	336.59	192.01	243.63	252.83	136.56	180.23	164.78
**30:1**	135.53	98.68	167.26	168.65	151.81	174.54	128.19	130.02	146.85	84.75	94.13	124.1
**15:1**	65.03	55.17	80.875	87.37	84.43	108.37	91.41	90.69	97.81	69.39	63.03	81.48
**7.5:1**	43.84	43.90	49.95	54.33	59.79	130.24	66.35	70.66	78.91	55.63	54.79	63.84
**3.8:1**	40.15	34.19	41.92	53.9	54.89	88.74	57.16	56.45	68.02	49.58	43.89	54.86
**1.9:1**	38.01	34.87	37.96	54.67	43.71	66.93	57.31	50.71	62.66	49.26	48.72	50.56
**ED Ratio (5-fold)**	25.1	24.7	21.2	75.1	59.6	53.3	118.1	65.3	97.3	93.5	81.8	111.7

Table S5. Comparative coagulation potency and antivenom neutralization efficiency across *Echis* venoms.

Sample	MCD	Mean	SEM	ED ratio (5fold)	Mean	SEM
ECS-BA	7.57	9.25	0.91	93.54	95.68	8.69
	10.70			81.81		
	9.46			111.68		
ECS-SA	7.28	8.02	0.37	75.11	62.68	6.48
	8.33			59.65		
	8.45			53.28		
ECS-PO	8.03	8.67	0.40	118.11	93.57	15.36
	8.59			65.29		
	9.39			97.32		
ECC-TN	3.75	3.80	2.19	25.10	23.68	1.22
	3.95			24.70		
	3.70			21.24		

Table S6. Post hoc Dunnett's multiple comparisons of effective dose (ED) ratios required for five-fold prolongation of clotting time using ECC-TN as the reference venom

Number of families=1; Number of comparisons per family=3; Alpha=0.05

Dunnett's multiple comparisons test	Mean Diff.	95% CI of diff.	Significant?	Summary	Adjusted P Value			
ECC-TN vs. ECS-BA	-71.9	-110.4 to -33.6	Yes	**	0.0017<br>0.0466			
ECC-TN vs. ECS-SA	-39.0	-77.4 to -0.6	Yes	*	0.0020			
ECC-TN vs. ECS-PO	-69.9	-108.3 to -31.5	Yes	**				
**Test details**	**Mean 1**	**Mean 2**	**Mean Diff.**	**SE of diff.**	**n1**	**n2**	**q**	**DF**
ECC-TN vs. ECS-BA	23.7	95.7	-71.9	13.3	3	3	5.4	8
ECC-TN vs. ECS-SA	23.7	62.7	-39.0	13.3	3	3	2.9	8
ECC-TN vs. ECS-PO	23.7	93.6	-69.9	13.3	3	3	5.2	8

Table S7: Details of the *Echis c. sochureki* venom samples collected from Rajasthan.

Specimen ID	Location	Latitude	Longitude	Snout Vent Length (cm)	Tail length (cm)	Total length (cm)	Sex	Ventral Scales	Sub-Caudal Scales	Mid Body	Venom yield volume	Estimated dry weight in mg (21-26 % of yield)
23.30	Chouhtan, Barmer	25.45985	71.05613	32.5	4.8	37.3	Female	175	32	31	NR	–
23.31	Kurja, Barmer	25.65963	71.38422	30	4.5	34.5	Female	171	30	31	NR	–
23.32	Chouhtan, Barmer	25.45997	71.05368	24.5	3	27.5	Female	182	34	27	NR	–
23.38	Sam-Kanoi road	26.85527	70.55278	27.5	2.9	30.4	Male	162	32	29	20 µl	4.7 ± 0.50
23.39	Sam desert	26.85949	70.451	56	6.2	62.2	Female	178	34	27	65 µl	15.25 ± 1.65
23.40	Sam desert	26.86507	70.44293	48.6	5.9	54.5	Female	172	32	29	100 µl	23.50 ± 2.50
23.41	Sam desert	26.86486	70.44323	55.5	6.3	61.8	Female	183	33	27	40 µl	9.40 ± 1.00
23.47	Chhilro, Sam, Jaisalmer	26.79318	70.36285	56	7	63	Female	177	39	27	200 µl	47.00 ± 5.00
23.48	Chhilro, Sam, Jaisalmer	26.79318	70.36285	41	4.4	45.4	Female	184	31	25	30 µl	7.05 ± 0.75
23.49	Chhilro, Sam, Jaisalmer	26.79407	70.36284	27.5	2.5	30	Female	160	28	28	10 µl	2.35 ± 0.25
23.50	Chhilro, Sam, Jaisalmer	26.79318	70.36285	53.7	7	60.7	Female	181	36	28	90 µl	21.15 ± 2.25
23.51	Chhilro, Sam, Jaisalmer	26.79336	70.36494	22	2	24	Female	126	41	32	15 µl	3.50 ± 0.40
23.52	Sam - Longewala highway, Jaisalmer	26.79229	70.36812	43	5	48	Female	158	33	28	30 µl	7.05 ± 0.75
23.53	Sam -Kanoi, Jaisalmer	26.82224	70.48613	55	5.5	60.5	Female	170	33	31	95 µl	22.30 ± 2.40

23.56	Sam resort, Jaisalmer	26.86339	70.5537	61	8.4	69.4	Female	196	32	33	115 µl	27.00 ± 2.90
23.57	Sam-Kanoi Road	26.86278	70.56217	62	7.5	69.5	Female	180	31	33	240 µl	56.40 ± 6.00
23.62	Kajoi, Phalsund, Pokhran	26.35898	71.88069	23.5	3.2	26.7	Female	178	35	29	NR	–
23.63	Phalsund, Pokhran	26.42114	71.89763	31.2	3.2	34.4	Female	182	34	23	NR	–
23.64	Phalsund, Pokhran	26.42013	71.89776	54	5.4	59.4	Female	173	32	33	NR	–
23.65	Phalsund, Pokhran	26.42268	71.90016	64.2	5.5	69.7	Female	183	33	31	NR	–
23.66	Phalsund, Pokhran	26.42052	71.8982	48.5	3.7	54.2	Female	179	27	29	NR	–
23.67	Ujla, Pokhran	26.82434	71.93674	37	4.3	41.3	Male	177	38	29	NR	–

NR- Venom yield volume was *not recorded* for these specimens.

The estimates of protein dry weight in the venom were derived in comparison with the dry solids range for viperid venoms reported by Mirtschin et al. [34].

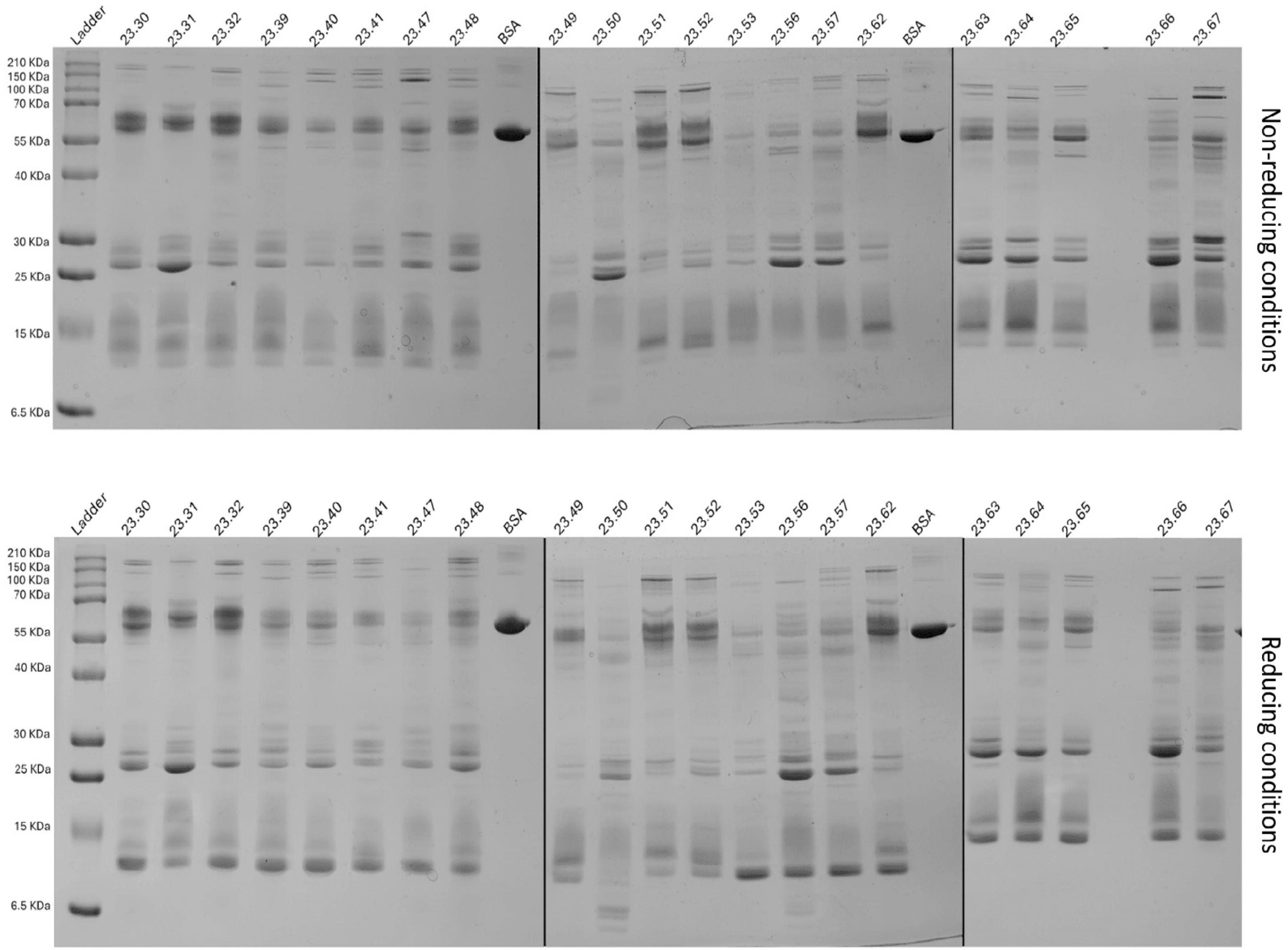

Figure S1. SDS-PAGE separation of *E. c. sochureki* venoms under reducing and non-reducing conditions.

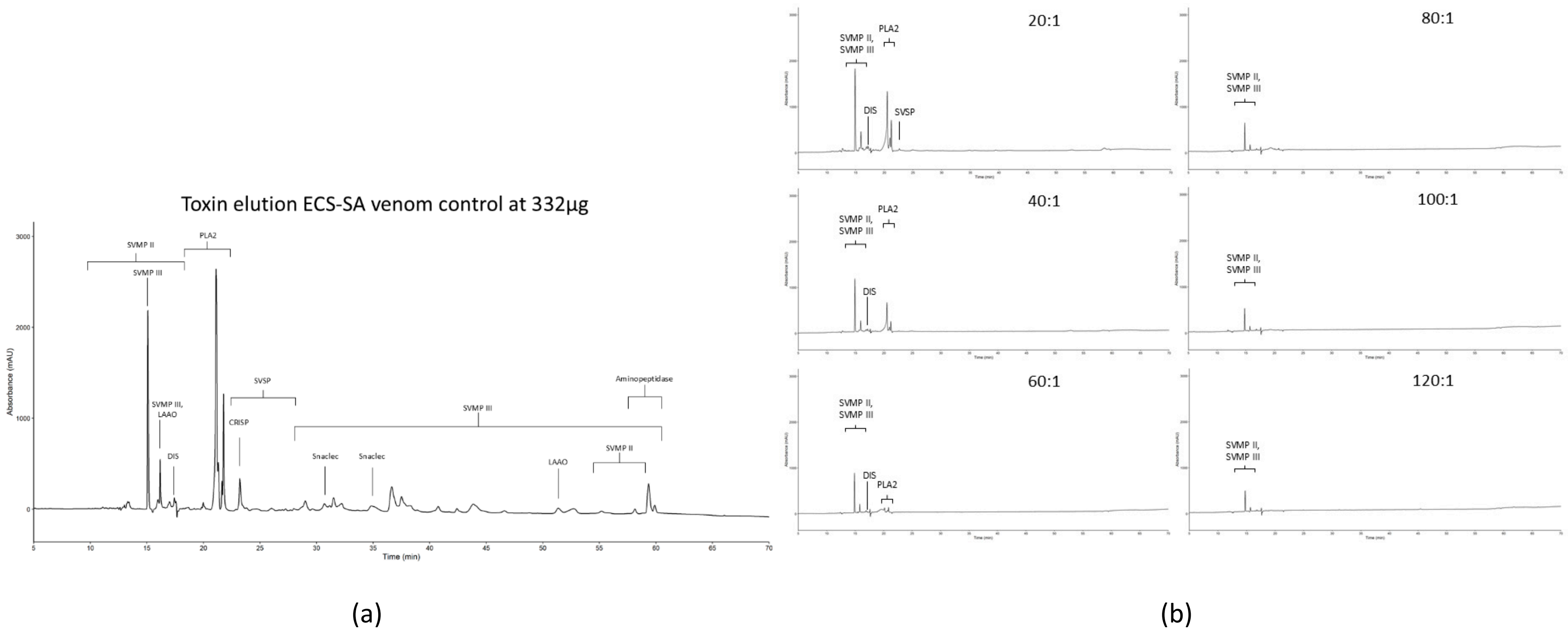

Figure S2. Reverse-phase HPLC profile of ECS-SA toxins after affinity chromatography. Panel A: Elution of toxins indicated for venom control at highest venom amount (332 µg). Panel B: Non retained toxins fractions at respective ratios after incubation with ~5 mg of IPAV. PLA2-Phospholipases; SP-Serine proteinases; P-II SVMP; P-III SVMP; Group I, II, III-Snake venom metalloproteinases; LAAO-L-amino acid oxidase.

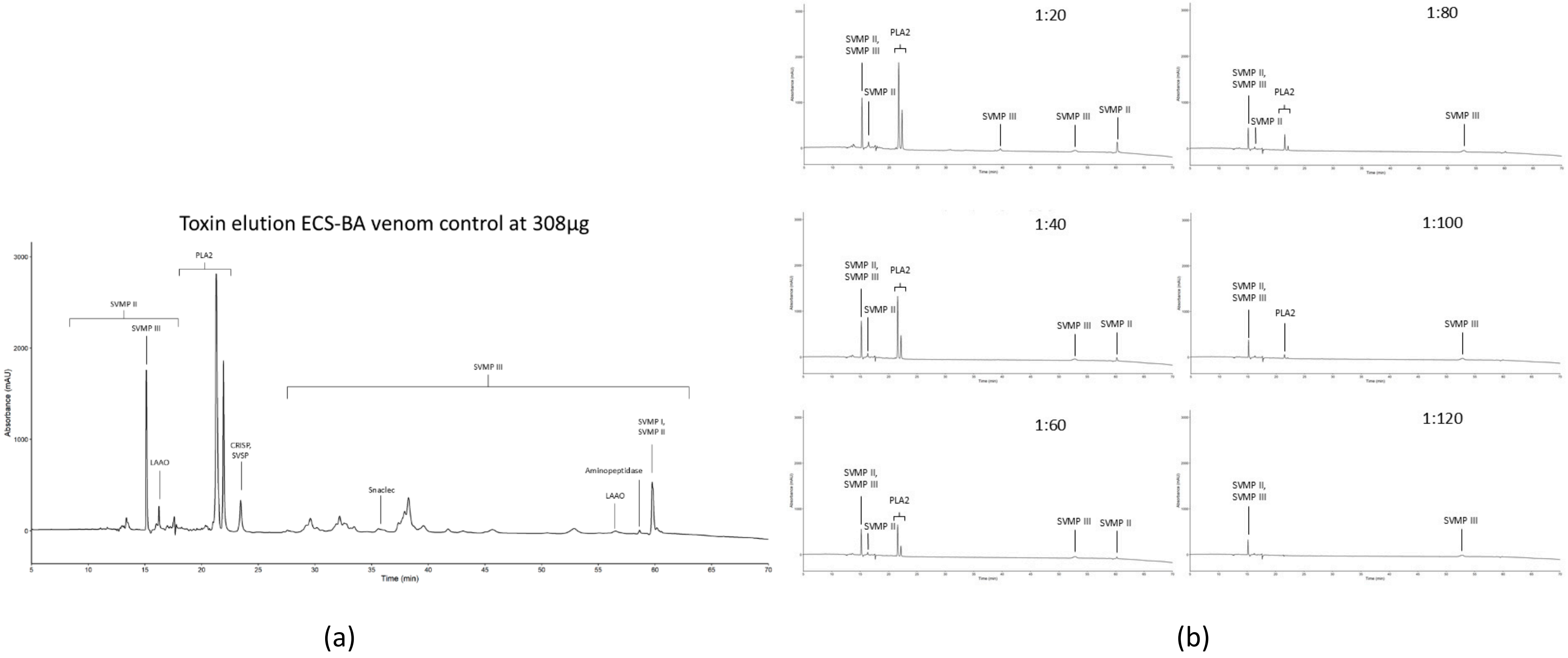

Figure S3. Reverse-phase HPLC profile of ECS-BA toxins after affinity chromatography. Panel A: Elution of toxins indicated for venom control at highest venom amount (308 µg). Panel B: Non retained toxins fractions at respective ratios after incubation with ~5 mg of IPAV.

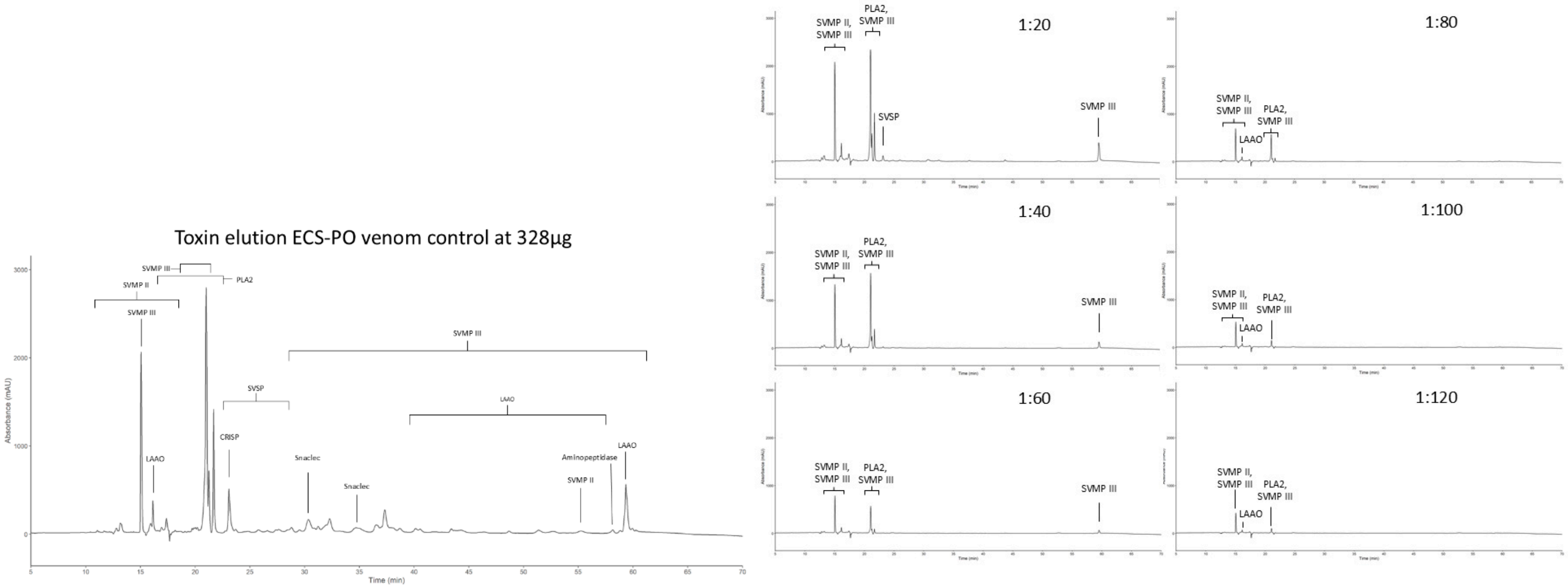

Figure S4. Reverse-phase HPLC profile of ECS-PO toxins after affinity chromatography. Panel A: Elution of toxins indicated for venom control at highest venom amount (328 µg). Panel B: Non retained toxins fractions at respective ratios after incubation with ~5 mg of IPAV.
